# A Compact Ultrawideband Antenna Based on Hexagonal Split-Ring Resonator for pH Sensor Application

**DOI:** 10.3390/s18092959

**Published:** 2018-09-05

**Authors:** Mohammad Tariqul Islam, Farhad Bin Ashraf, Touhidul Alam, Norbahiah Misran, Kamarulzaman Bin Mat

**Affiliations:** Centre of Advanced Electronic and Communication Engineering, Universiti Kebangsaan Malaysia, Bangi, Selangor D.E. 43600, Malaysia; farhadbinashraf@siswa.ukm.edu.my (F.B.A.); touhid13@siswa.ukm.edu.my (T.A.); bahiah@ukm.edu.my (N.M.); kamarulzaman@ukm.edu.my (K.B.M.)

**Keywords:** double negative, metamaterial, pH sensor, split-ring resonator, ultrawideband

## Abstract

A compact ultrawideband (UWB) antenna based on a hexagonal split-ring resonator (HSRR) is presented in this paper for sensing the pH factor. The modified HSRR is a new concept regarding the conventional square split-ring resonator (SSRR). Two HSRRs are interconnected with a strip line and a split in one HSRR is introduced to increase the electrical length and coupling effect. The presented UWB antenna consists of three unit cells on top of the radiating patch element. This combination of UWB antenna and HSRR gives double-negative characteristics which increase the sensitivity of the UWB antenna for the pH sensor. The proposed ultrawideband antenna metamaterial sensor was designed and fabricated on FR-4 substrate. The electrical length of the proposed metamaterial antenna sensor is 0.238 × 0.194 × 0.016 λ, where λ is the lowest frequency of 3 GHz. The fractional bandwidth and bandwidth dimension ratio were achieved with the metamaterial-inspired antenna as 146.91% and 3183.05, respectively. The operating frequency of this antenna sensor covers the bandwidth of 17 GHz, starting from 3 to 20 GHz with a realized gain of 3.88 dB. The proposed HSRR-based ultrawideband antenna sensor is found to reach high gain and bandwidth while maintaining the smallest electrical size, a highly desired property for pH-sensing applications.

## 1. Introduction

Microwave sensors using metamaterial antennas have great potential in the field of sensors because of their simplicity, portability, sensitivity, and cost effectiveness. Water plays an important role in the development of civilization. Nowadays, protection of human health is an alarming concern for researchers. For many biochemical industries like food, cosmetics, and beverages, pH factor is a vital parameter to monitor. The number of water-related diseases and deaths due to lack of pure water is increasing daily [1]. The measurement of water quality depends on several variables, including conductivity [2] and pH [3]. Each of them has a different range of acceptable values depending on the application. Available standard methods that focus on the features and characterization of various kind of pH sensors are presented in [4]. The solid-state reference electrode (SSRE) [5] is a popular pH sensor that minimizes the ongoing problems with conventional reference electrodes. In recent years, there has been a huge interest in metamaterial antenna sensors for determination of the pH value of liquid since ultrawideband (UWB) metamaterial antennas offer good penetration and resolution characteristics. Ultrawideband antennas have been used for near-field imaging [6], UWB radar sensors [7], temperature sensors [8], and indoor identification and localization systems [9]. State-of-the-art metamaterial-based UWB antenna sensors have been considered eminent among other microwave pH sensors.

In 1968, Victor Veselago first introduced a theoretical explanation of a material which has unique electromagnetic properties that cannot be found in nature [10]. The unique properties of this material are that it can simultaneously give negative permittivity and permeability. Thirty years later, Smith et al. introduced an artificial material by using a split-ring resonator and metallic wire that shows negative permittivity and permeability at the same time [11]. These artificially structured composites have the potential to fill the void in the electromagnetic field. The impedance of metamaterials can be matched to the impedance of free space by alteration of permittivity (*ε*) and permeability (*µ*). In this context, researchers have developed and investigated the extraordinary properties of metamaterials and have come up with many designs, such as complementary split-ring resonators [12], elliptical split-ring resonators [13], omega-shaped metamaterials [14], and so on. Due to their unique electromagnetic properties, they can be used in invisibility cloaks [15], gain enhancement [16], filters [17], sensors [18], highly directive subwavelength cavity antennas [19,20,21], etc. Metamaterial structures have been adopted by many researchers because of their cost effectiveness, size reduction, and label-free detection. A proper geometry of metamaterials can control the sensitivity of the sensor to both electric and magnetic fields and their working resonant frequency [22]. Lee et al. [12] presented a complementary split-ring resonator (CSRR) microwave sensor for the S-band (2–4 GHz) frequency to detect the variation in terms of permittivity or thickness. The changes in transmission coefficient are observed for multilayer dielectric structures. A compact polarization-insensitive dual-band metamaterial absorber for the X-band (8–12 GHz) was proposed in [23]. There, they used a 0.6-mm FR-4 epoxy resin dielectric substrate to make it ultrathin. Alam et al. [24] proposed a hexagonal metamaterial structure that achieved double-negative characteristics of about 1.50 and 0.95 GHz for mobile wireless communication systems. Zhou et al. [25] proposed a multiband left-handed metamaterial (LHM)-based double Z-shaped resonator. The structure exhibits three passbands at 7.3, 8.1, and 9.4 GHz. However, the double-sided structure makes it difficult to fabricate.

Microwave sensing technology has been developed over the years for several sensing applications, including liquid solution concentrations [26], moisture sensors [27], and real-time glucose monitoring [28]. Microstrip patch antennas are widely used in sensing applications [29,30,31]. The main advantages of patch antennas are their light weight, small size, low fabrication cost, reliability, and durability. Patch antennas as microwave sensors work by interacting the electromagnetic waves and dielectric properties. The interaction results in a change in frequency, which can be related to the nature of the sample being tested. Rodrigues et al. [32] proposed a low-cost microwave radiometric sensor for noninvasive detection of brain temperature. A 25 × 28 mm^2^ microstrip log-spiral antenna was used as a sensor element. The authors in [33] proposed an UWB radio sensor for monitoring elderly people who live alone. The functional frequency band is from 6 to 8.5 GHz and it also monitors position and breathing motion. A rectangular-shaped UWB antenna was reported in [34] for breast cancer detection. The antenna size was 36 × 34 mm^2^ with an operating frequency from 2.68 to 12.06 GHz, which is still large. A combination split-ring resonator (SRR) and capacitive-loaded strip (CLS) UWB antenna was reported in [35] with dimensions of 0.21 × 0.20 × 0.015 λ for 2.90 GHz. However, the antenna does not cover the UWB frequency range and is only able to manage from 2.9 to 9.9 GHz. A configurable meta-inspired UWB monopole antenna was presented in [36] with a maximum peak gain of 6 dBi and maximum efficiency of 70%. Four Ω-shaped strip layers were used in the SRR to make it configurable. In [37], a compact ultrawideband antenna was investigated. The antenna consists of a spectacle-shaped resonator and a tapered-slot ground plane with a radiation efficiency of 89% and an average gain of 5.7 dBi. A negative index metamaterial-inspired UWB antenna that integrates complementary SRR and CLS unit cells has been presented for microwave imaging sensor applications [38]. The UWB antenna sensor has an electrical dimension of 0.20 × 0.29 λ and achieves a 131.5% bandwidth, covering the frequency bands from 3.1 to 15 GHz with a maximum gain of 6.57 dBi. The antenna has a high fidelity factor and nearly omnidirectional radiation patterns with low cross polarization, which are suitable for microwave imaging. In [39], a UWB antenna with the smallest form factor was reported for breast tumor sensing applications, utilizing two rectangular split-ring resonators (RSRR) and four rectangular complementary split-ring resonators (RCSRR). The proposed antenna delivers a wide impedance bandwidth from 3 to 11 GHz with nearly omnidirectional radiation patterns and good radiation efficiency over the entire frequency band. In [40], they designed a compact quad-notched UWB antenna by utilizing CSRRs on the radiating semicircular patch for rejecting the WiMAX, INSAT, and lower and upper WLAN bands. They also analyzed the coupling among the multiple-notch resonators due to coupling near CSRRs.

In this paper, a miniature UWB metamaterial antenna based on a hexagonal split-ring resonator (HSRR) was designed and analyzed for sensing the pH factor of liquid. The hexagonal metamaterial unit cell exhibits a wide double-negative characteristic from 7.43 to 11.79 GHz. Moreover, an array configuration of the proposed UWB antenna was also investigated to see the response to variations in the number of unit cells. The proposed UWB antenna consists of three hexagonal split-ring resonators along with a slotted elliptical patch and a microstrip feed line. A partial ground plane was also used, which was rectangular and elliptically slotted to achieve the targeted frequency band. The antenna had a wide UWB profile along with high gain and efficiency, stable radiation patterns, and electrical dimensions of 0.238 × 0.194 × 0.016 λ. This article is ordered as follows. Section 2 describes the configuration of the metamaterial structure, extraction method, and discusses the results. The scattering parameters of the unit cell and UWB antenna was obtained by using the CST Microwave Studio based on the finite integration technique (FIT) method. In Section 3, the UWB metamaterial antenna sensor configuration, gain, radiation patterns, and efficiency are presented. Section 4 describes the sensing of pH for different solutions. Finally, Section 5 presents the conclusion.

## 2. Hexagonal Split-Ring Resonator

### 2.1. Materials and Methods

**Figure 1:**
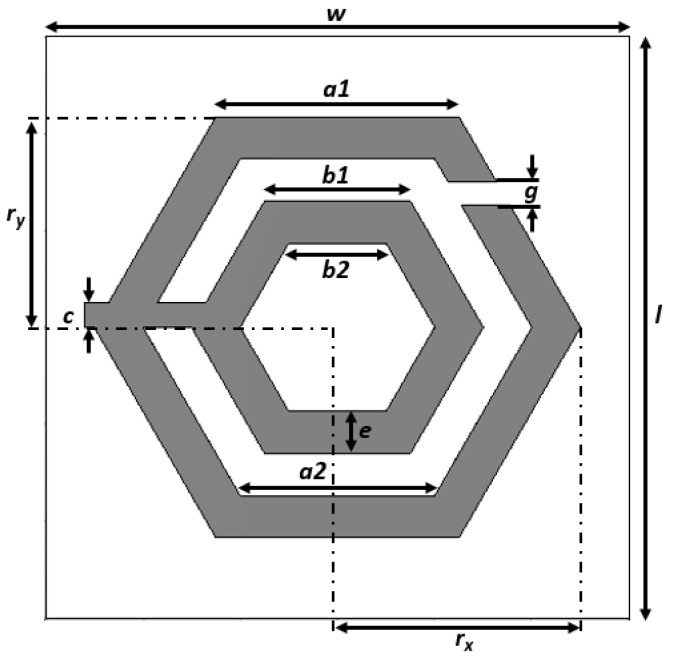
Proposed hexagonal split-ring resonator (HSRR) structure of the unit cell.

The configuration of the proposed unit cell is shown in Figure 1. The metamaterial structure consisted of two hexagonal split-ring resonators interconnected by a strip line. The FR-4 (*ε*_r_ = 4.3, *µ*_r_ = 1, and *δ* = 0.025) substrate was employed and the overall dimensions of the structure were 6 × 6 × 1.6 mm^3^. The conductive copper layer of the substrate was 0.035 mm thick. The design parameters of the unit cell are represented in Table 1.

**Figure 2:**
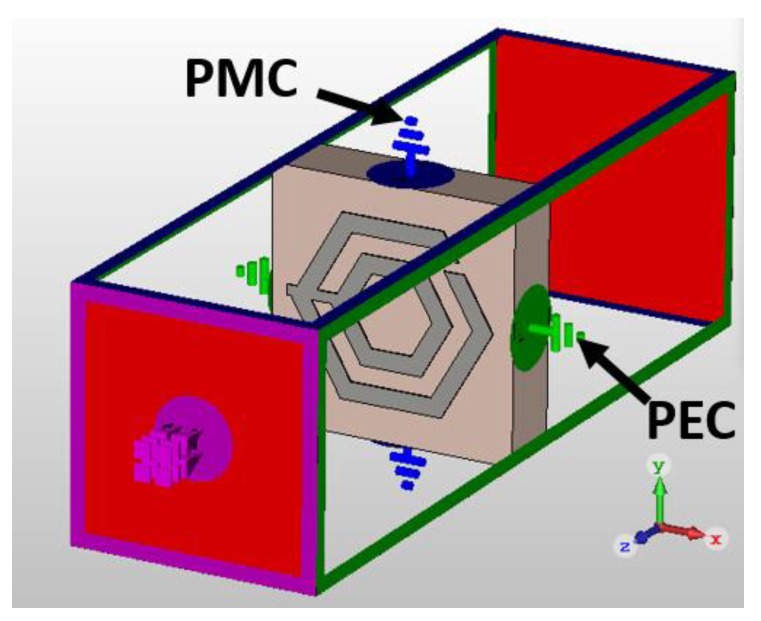
Simulation setup of the proposed HSRR structure.

The S-parameters of the unit cell were obtained by using the CST Microwave Studio based on the finite integration technique (FIT) method. The transverse electromagnetic (TEM) mode was used to investigate the metamaterial performance. Perfect electric conductor (PEC) in the x-direction and perfect magnetic conductor (PMC) in the y-direction were set as boundary conditions. Throughout the z-direction, the electromagnetic wave was propagated. The simulation setup of the unit cell is shown in Figure 2. The effective parameters of the proposed unit cell were extracted by using the transmission-reflection method [41].

(1) $$S_{11}=\frac{R_{01}(1-e^{i2nk_{0}d})}{1-R^{2}{}_{01}e^{i2nk_{0}d}}$$

(2) $$S_{21}=\frac{(1-R^{2}{}_{01})e^{ink_{0}d}}{1-R^{2}{}_{01}e^{i2nk_{0}d}}$$

where $R_{01}=z-1/z+1$

$$e^{ink_{0}d}=\frac{s_{21}}{1-s_{11}R_{01}}$$

The refractive index *η* and the impedance *z* are obtained by

(3) $$z=\pm \sqrt{\frac{{\left(1+S_{11}\right)}^{2}+S^{2}{}_{21}}{{\left(1-S_{11}\right)}^{2}+S^{2}{}_{21}}}$$

(4) $$\eta =\frac{1}{k_{0}d}\cos^{-1}\left[\frac{1}{2S_{21}}(1-S^{2}{}_{11}+S^{2}{}_{21})\right]$$

(5) $$\varepsilon =\frac{\eta }{z}$$

(6) μ=ηz

where *k*_0_ = ω/*c* is the free-space wavevector, ω is the angular frequency, and *c* is the speed of light. Symbol *ε* is the effective permittivity, *μ* is the effective permeability, and *d* denotes the thickness of the substrate material.

### 2.2. Chracterization of HSRR

**Figure 3:**
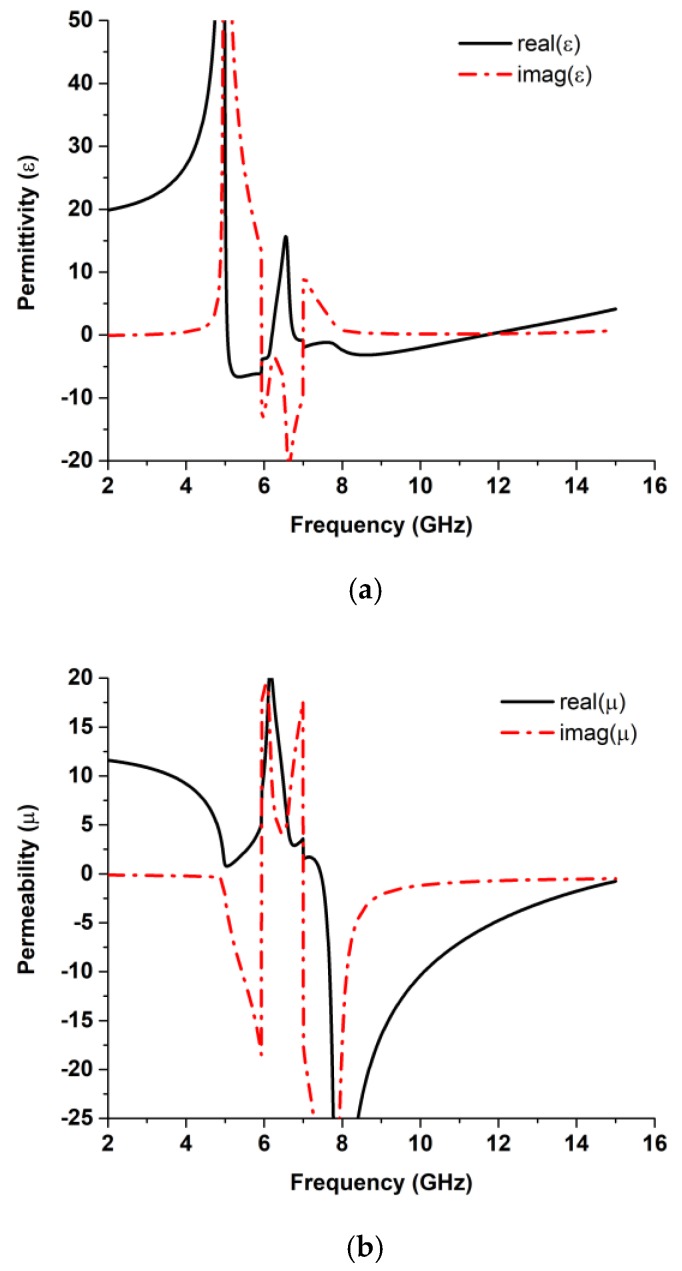

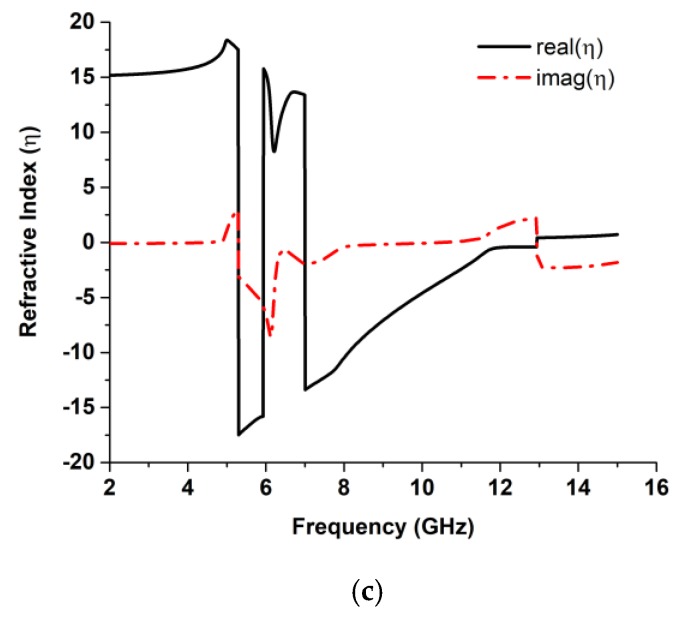
Effective Parameters: (**a**) permittivity, (**b**) permeability, and (**c**) refractive index.

The real and imaginary parts of the effective permittivity, effective permeability, and refractive index parameters are illustrated in Figure 3 to demonstrate that the proposed HSRR has negative permittivity from 5 to 6.21 GHz and also from 6.75 to 11.79 GHz and negative permeability over the frequency band from 7.43 to 15 GHz. The plasma frequency of the structure can be varied by changing the dimensions of the wire length. The structure has a double-negative characteristic bandwidth of 4.36 GHz. The variations in the value of the effective parameters depend on the values of S_11_ and S_21_ according to Equations (1)–(6). A summary of the effective parameters is listed in Table 2.

## 3. UWB Metamaterial Antenna

### 3.1. Boundary Condition

The electromagnetic field interacts with the metallic inclusions of the metamaterial, the metallic inclusions placements, and their distributions. Therefore, the electric and magnetic fields are oriented in a specific direction, which retrieves the permittivity and permeability characteristics. The response of the metamaterial depends on the direction of the magnetic field, which is normal to the surface of the structure, and the electric field is tangent to the inclusions. The precise polarization and distribution of the electromagnetic field must be specified by the characterization technique. In this article, as the PEC boundary condition was applied at the upper and lower walls of the HSSR unit cell that was perpendicular to the incident **E** vector, the PMC boundary was applied to the back and front walls of the HSSR that was perpendicular to the incident **H** vector. For antennas operating in free space, the open add space boundary condition was used. To record the far-field pattern, this boundary condition was essential. Furthermore, to investigate the electric field in the radiating near-field region, the added space was manually extended in the open add space boundary conditions by a distance of 2D^2^/λ, where D is the diameter of the antenna and λ is the free space wavelength. Therefore, by adding specific fields like electric, magnetic, and open space at every wall, the field characteristics for resonant frequencies can be investigated.

### 3.2. Configaration of UWB Antenna

**Figure 4:**
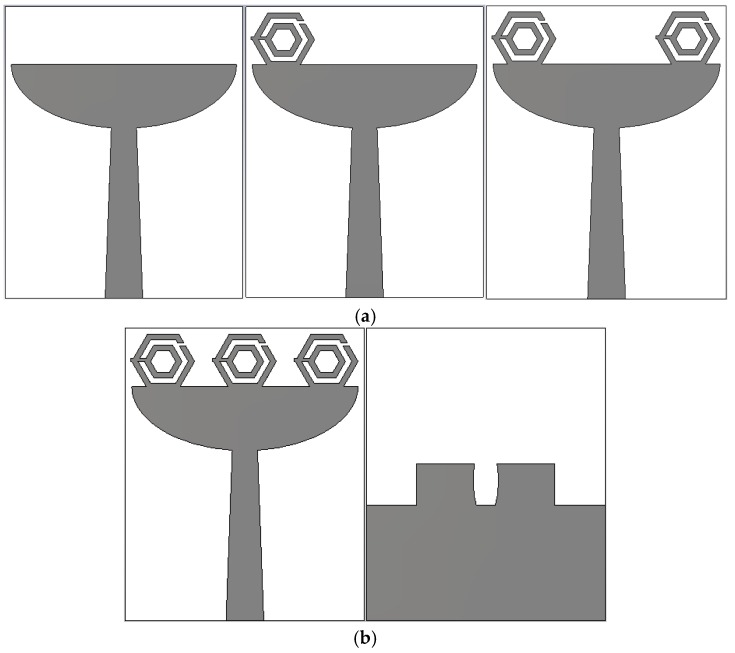
Ultrawideband (UWB) metamaterial antenna with (**a**) design evolution and (**b**) final design.

**Figure 5:**
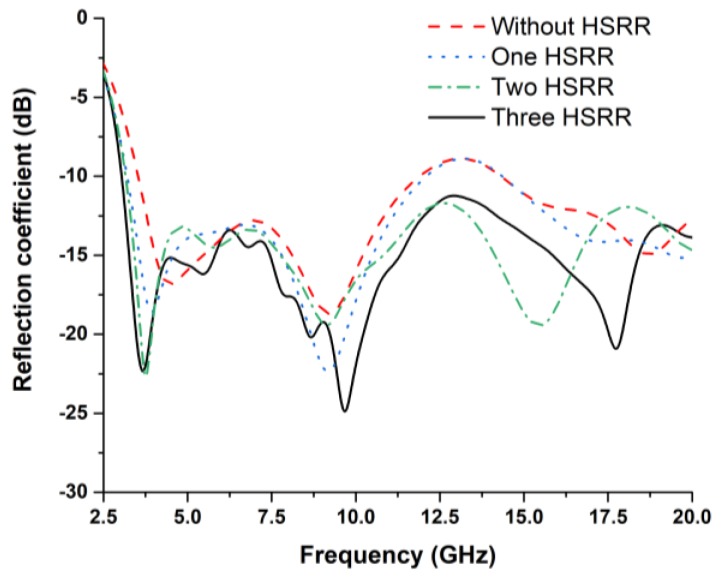
Reflection coefficient of UWB metamaterial antenna without and with HSRR.

The antenna was investigated without HSRR and by using one, two, and three hexagonal split-ring resonators, as shown in Figure 4a,b. The unique electromagnetic properties of the HSRR were responsible for the miniaturization of the antenna. The reflection coefficient (S_11_) of these configurations was studied. From Figure 5, S_11_ of the antenna without HSRR and with one HSRR shows a wide bandwidth response (below −10 dB), but the Federal Communication Commission (FCC) standard for a UWB antenna is not fully covered. The antenna with two HSRRs showed ultrawideband response, but with the three HSRR, a better reflection coefficient was obtained. Therefore, the antenna with three HSRRs was used as the final prototype.

**Figure 6:**
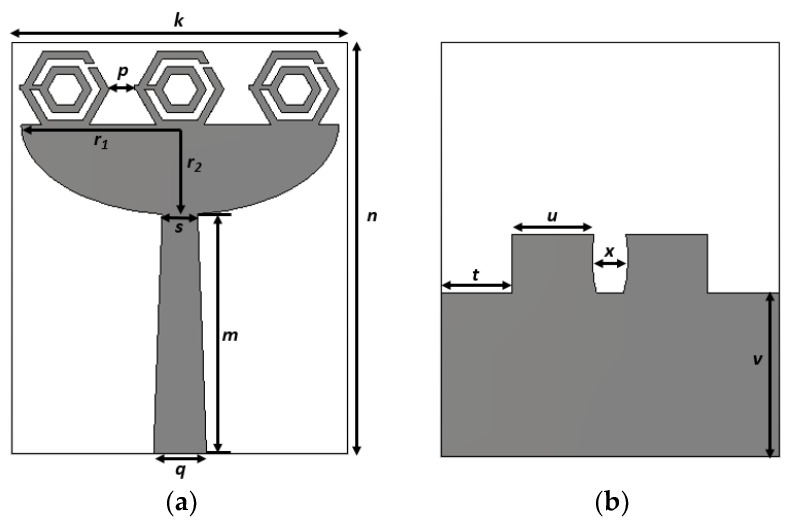
Proposed UWB metamaterial antenna. (**a**) Front view and (**b**) back view.

The configuration of the proposed UWB metamaterial antenna is shown in Figure 6. The three hexagonal split-ring resonators were on top of a slotted elliptical patch. A microstrip feed line was used to feed the antenna. A partial ground plane was rectangular and elliptically slotted in the middle to achieve UWB frequency band. The antenna was fabricated on a flame-resistant composite material named FR-4 (*ε*_r_ = 4.3, *µ*_r_ = 1, and *δ* = 0.025) substrate. The overall dimensions of the UWB antenna were 19 × 23.35 × 1.6 mm^3^. The conductive copper layer of the substrate was 0.035 mm thick. The design parameters of the proposed antenna are represented in Table 3.

### 3.3. Results and Discussion of UWB Antenna

**Figure 7:**
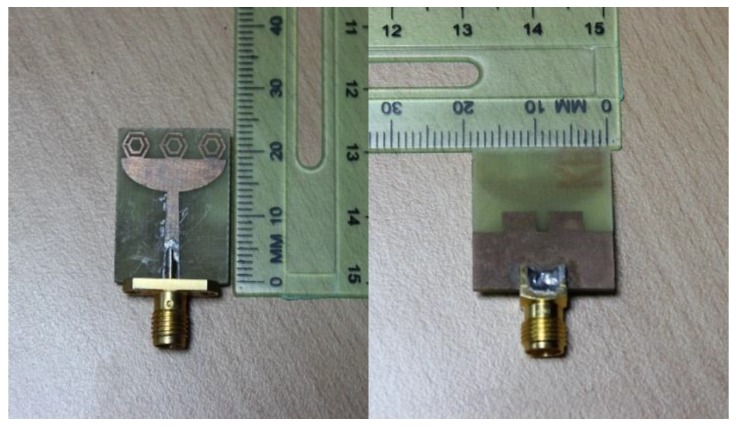
Fabricated prototype of the designed UWB antenna.

**Figure 8:**
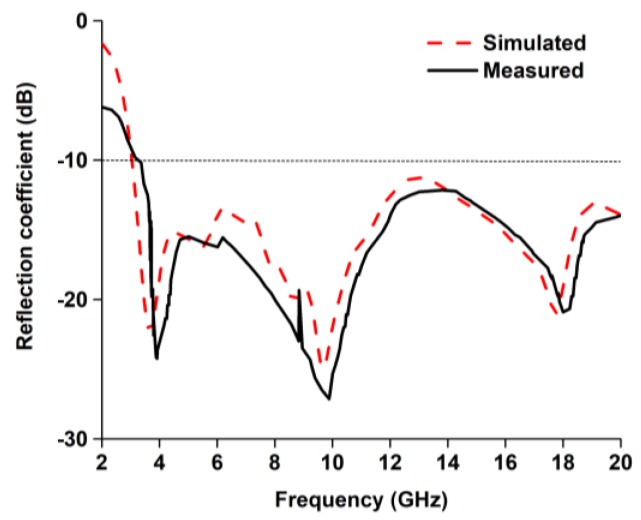
Simulated and measured reflection coefficient of the UWB antenna.

A prototype of the proposed UWB antenna was designed and fabricated according to the optimized parameters in Table 3 and as depicted in Figure 7. The computational and measured reflection coefficients of the antenna are plotted in Figure 8, which show very good agreement between them. The measurement was performed using Agilent performance network analyzer N5227A. The measured −10 dB reflection coefficient bandwidth was from 3 to 20 GHz. The antenna compactness with wideband characteristics can be expressed in terms of the bandwidth dimension ratio (BDR) [42]. The bandwidth dimension ratio of the proposed antenna was shown to be 3183.05 by using Equation (7).

(7) $$BDR=\frac{\left(BW\%\right)}{\lambda_{length}\times \lambda_{width}}$$

**Figure 9:**
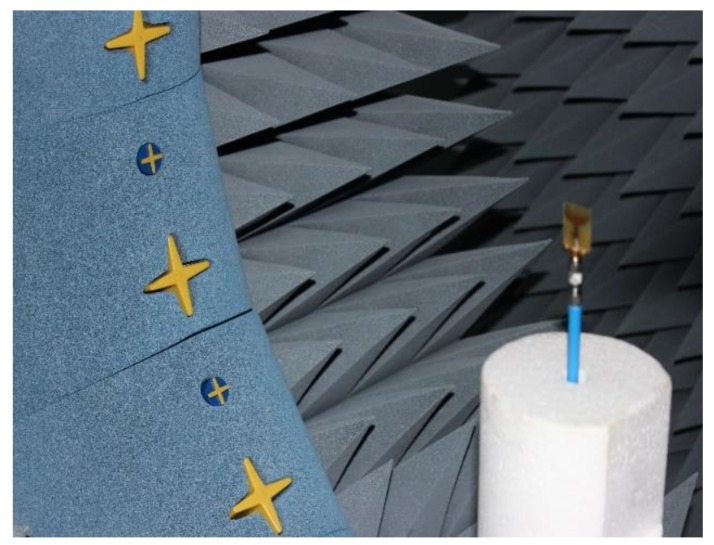
Antenna radiation characteristics measurement setup.

**Figure 10:**
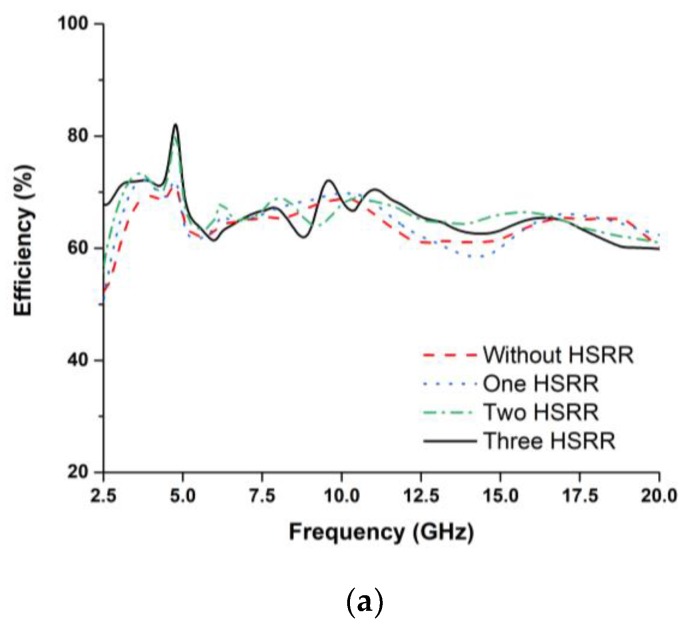

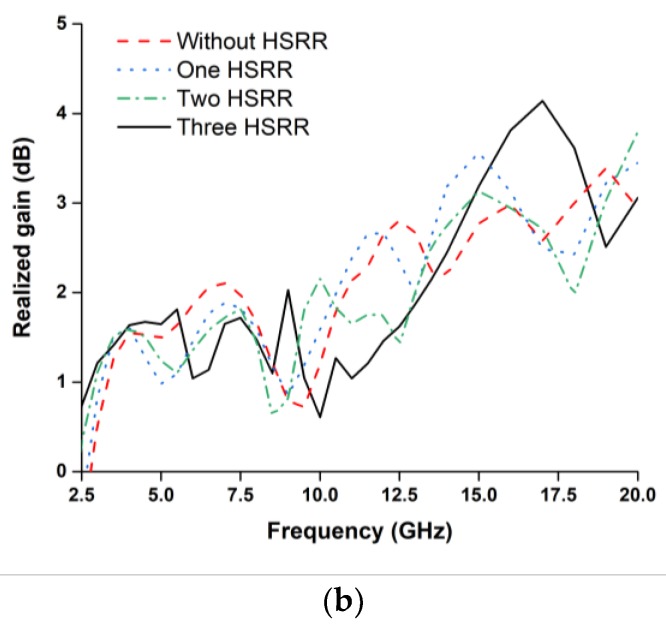
Simulated (**a**) efficiency and (**b**) realized gain of the UWB antenna for different orientations.

**Figure 11:**
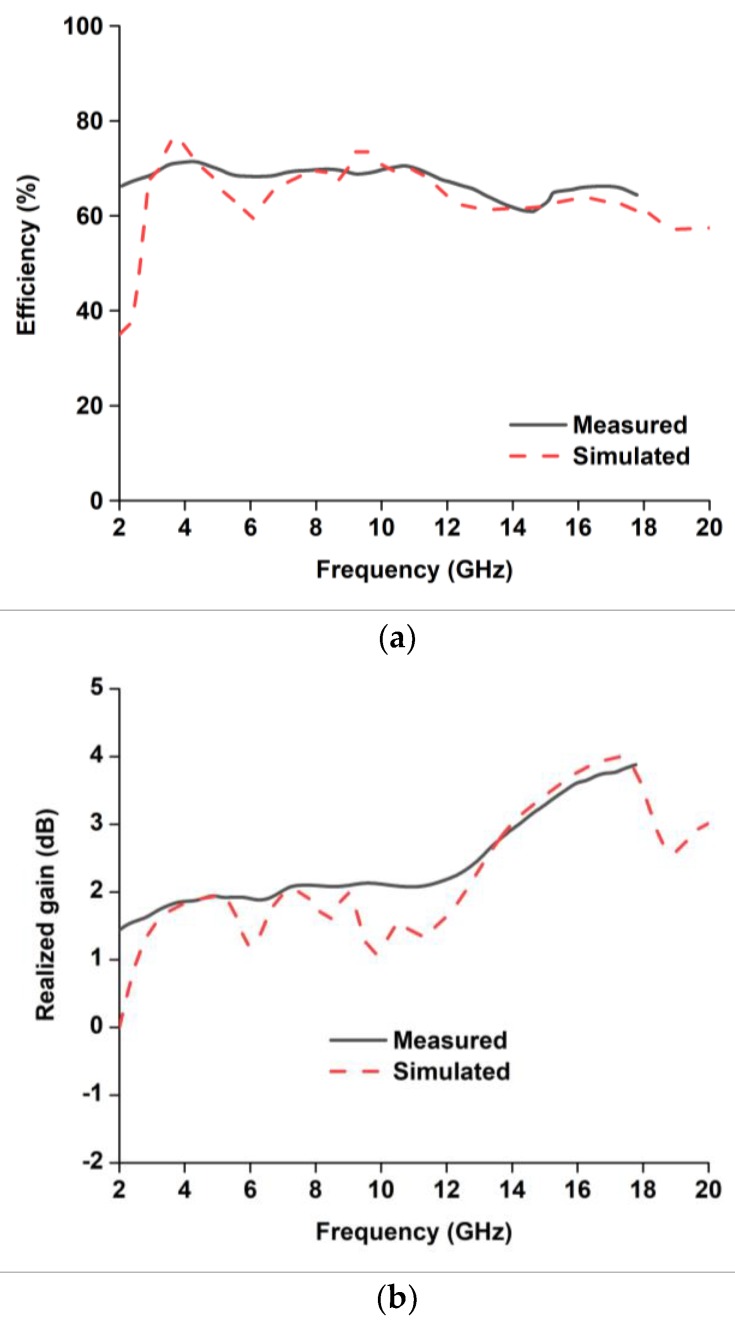
Simulated and measured (**a**) efficiency and (**b**) gain of the UWB antenna.

The radiation performances of the fabricated antenna were measured using a SATIMO near-field measurement system, illustrated in Figure 9. Figure 10a,b shows the antenna total efficiency and realized gain as a function of frequency for without and with HSRR. The measured and simulated efficiency and realized gain shows good agreement despite a small discrepancy due to fabrication and measurement tolerances. The average measured efficiency was found to be about 70% and the realized gain is plotted in Figure 11a,b. The average realized gain between 2 and 12 GHz is about 2 dB. Moreover, the gain increases up to 3.88 dB at the higher frequency of 15 GHz, where the radiation pattern becomes directional with some nulls due to the excitation of the higher-order current mode.

**Figure 12:**
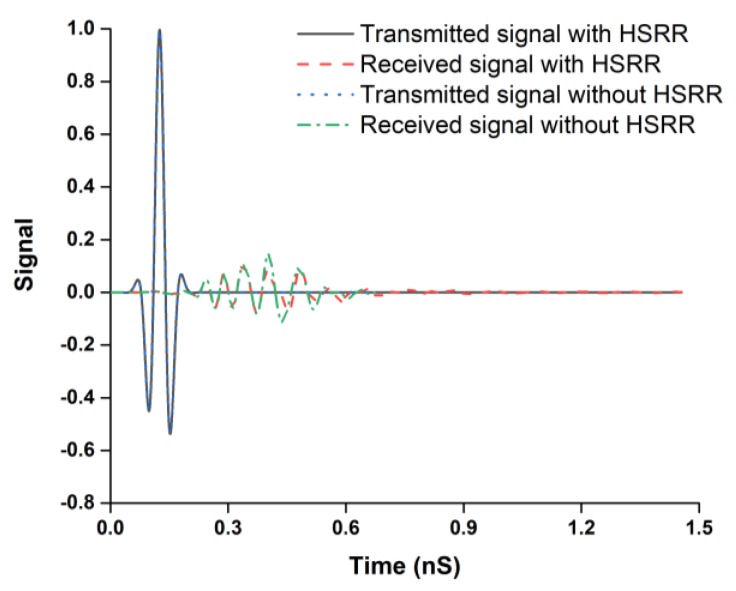
Transmitted and received signals without and with HSRR.

For dispersion analysis in the time domain, the antenna is derived by the input signal. The transmitted and received signal without and with HSRR is illustrated in Figure 12. From the pulse shapes, it can be observed that the transmitted pulse is not considerably distorted. The correlation coefficient between the received and transmitted signals can demonstrate the amount of pulse distortion that the antenna induced [43].

**Figure 13:**
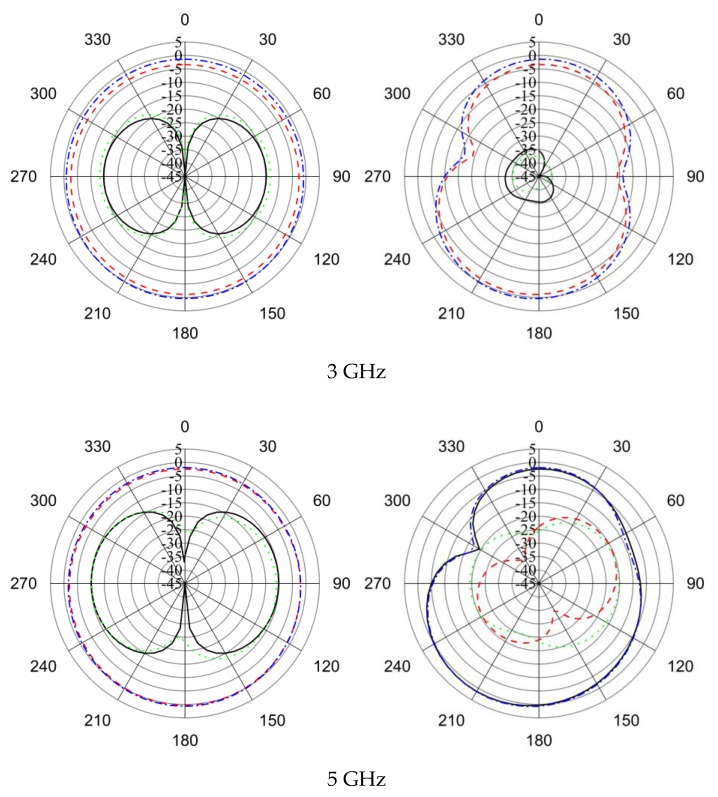

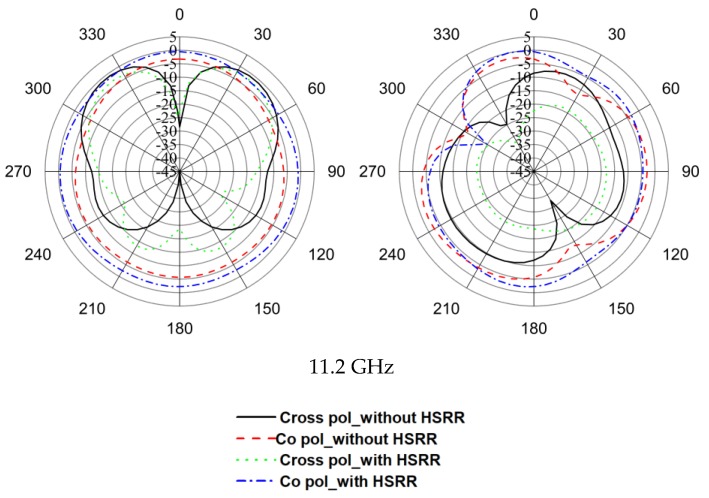
2D radiation pattern of the UWB antenna without and with HSRR.

The radiation pattern without and with HSRR at different frequencies is illustrated in Figure 13. The antenna achieved an omnidirectional radiation pattern over the whole bandwidth.

Phi = 0       Phi = 90

**Figure 14:**
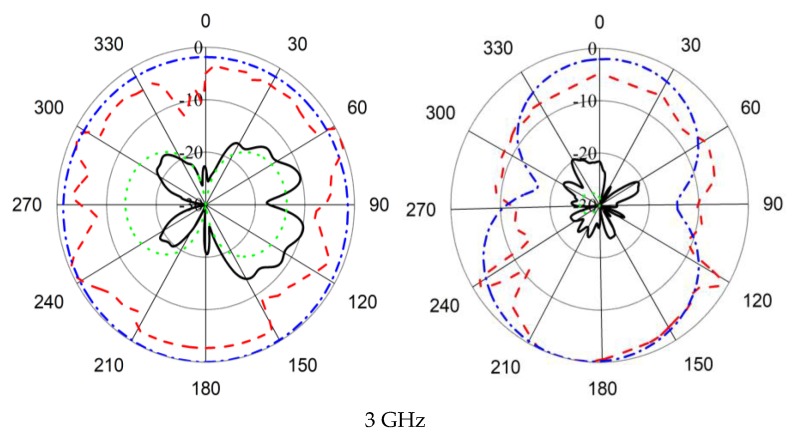

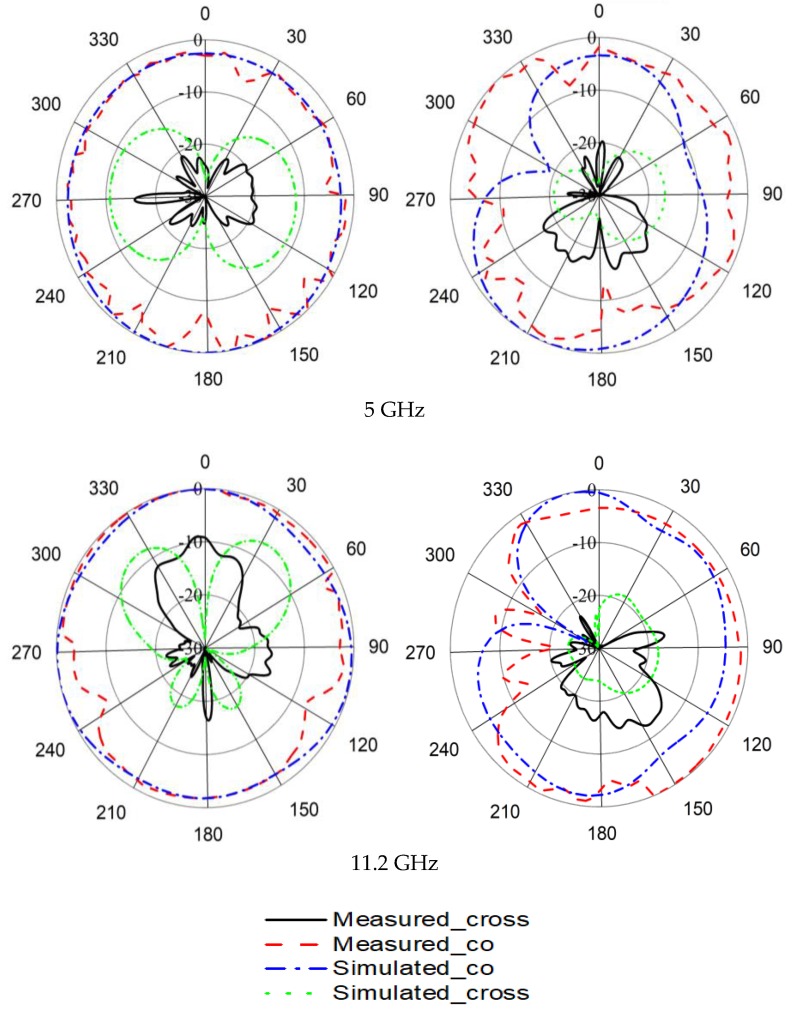
Simulated and measured radiation patterns of the proposed UWB antenna.

From Figure 14, it can be observed that the simulated and measured radiation patterns of the proposed antenna are in good agreement. Cross polarization can be negligible at 2.91 GHz both in the simulated and measured results. In the measured results, cross polarization is lower than simulated radiation patterns. The occurrence of slight disagreement between measured and simulated radiation patterns is due to fabrication and measurement tolerance.

Phi = 0       Phi = 90

To compare the proposed metamaterial-inspired UWB antenna sensor with existing antennas reported in the literature, a comparison is presented in Table 4.

## 4. UWB Antenna as pH Sensor

The fundamental principle of metamaterial-based microwave sensors depends upon the dielectric perturbation phenomenon. Therefore, the microwave sensor is used to measure the transmission coefficient of the sensor within different concentrated acid and base solutions. The amplitude of the S-parameters changes because of the change in pH of the solutions. The dielectric constant decreases with the increase in pH level in the solution for high frequency [44]. This is due to the positive and negative ions of the solution. The more concentrated the solution, the closer the positive and negative ions. When these two opposite ions come closer, the retardation force increases. Hence, the ions face greater resistance. As a result, the dielectric properties change due to the bond of dissolved ions and water molecules when acids and bases are added to the water. This change reduces the polarization of water and decreases the dielectric constant and loss factor [45].

The sensitivity of the microwave pH sensor was investigated with different pH standard solutions by using the N5227A Performance Network Analyzer. The solution was prepared by mixing sodium hydroxide (NaOH) and hydrochloric acid (HCL) with various volume ratios. To measure the pH level of the solutions, a pH meter having a measure range from 0 to 14 pH was used. The pH meter was dipped into the solutions until the potential reached an equilibrium value. Nine solutions with pH levels from 2.5 to 12.11 were used and the corresponding S-parameters were measured. The metamaterial antenna sensor was sensitive to the pH level based on the dielectric properties and conductivity of the solutions in terms of the transmission coefficient.

**Figure 15:**
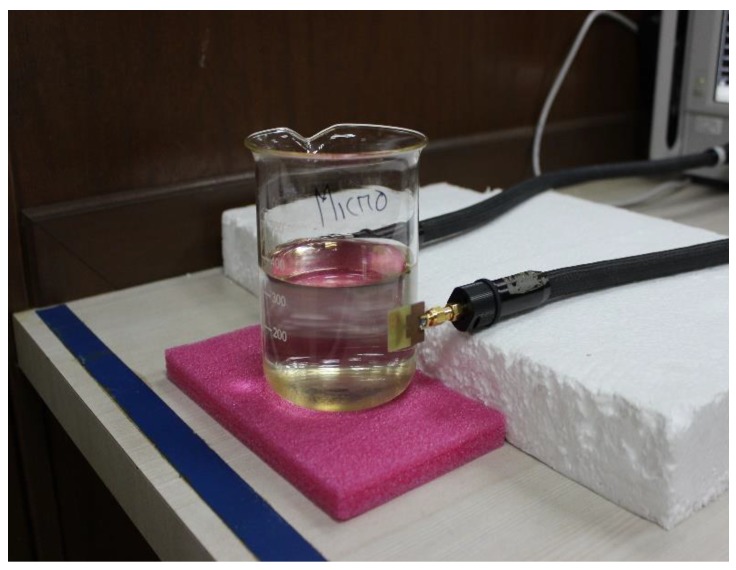
Measurement setup of the proposed UWB antenna sensor.

**Figure 16:**
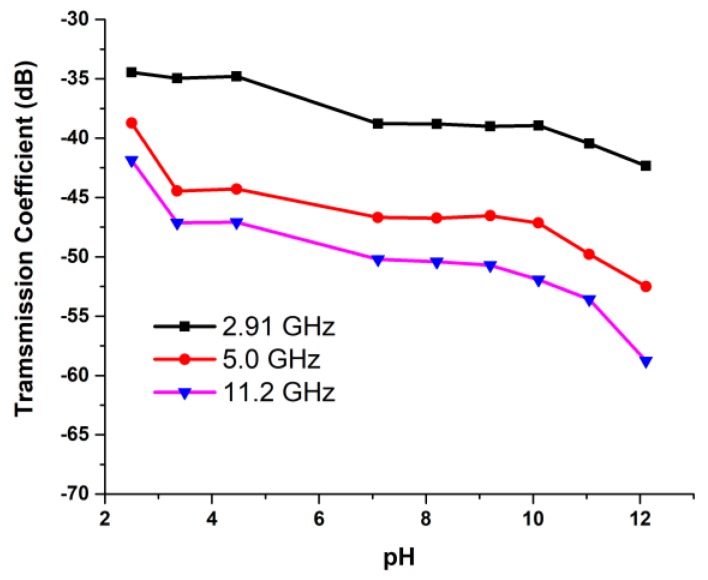
Measurement setup of the proposed UWB antenna sensor.

The sensor investigated the various concentrated solutions of NaOH and HCL. The tested water sample had a pH level of 7. First, the base substance NaOH was added to increase the pH of water and, secondly, the acidic substance HCL was added to decrease the pH of water. The reflection and transmission coefficients of the metamaterial antenna sensor were measured by placing the solutions between the two antennas. The measured arrangement is depicted in Figure 15. The sensor detected the pH of the solutions at 2.91, 5, and 11.2 GHz. The frequency response of the sensor antenna was plotted against pH values, as shown in Figure 16. From Figure 16, it can be clearly observed that the transmission coefficients increase while increasing the pH of the acid and base solution. The reduction of the permittivity of the solution caused the reduction of the effective dielectric constant of the solution. Due to the reduction of the effective dielectric constant of the solution, the load impedance increased. Hence, the transmission coefficients gradually increased with the increase of pH.

## 5. Conclusions

A compact ultrawideband antenna based on a hexagonal split-ring resonator has been presented with two interconnected HSRRs and a split in one HSRR for the miniaturization, increase in electrical length, and coupling effect for pH sensor application. The proposed sensor antenna has increased ultrawideband performance (3–20 GHz) while reducing the total dimensions by inserting the metamaterial unit cell. The UWB antenna sensor shows the fractional bandwidth and bandwidth dimension ratio of 146.91% and 3183.05, respectively. The total efficiency of the presented sensor antenna is about 70% with 3.88 dB realized gain. The proposed antenna managed to reach high gain and bandwidth while maintaining the smallest electrical size, which is a highly desired property for pH sensing applications.

## Figures and Tables

**Table 1:** Design parameters of the unit cell.

Parameters	Values mm	Parameters	Values mm
***a1***	2.5	***g***	0.3
***a2***	2	***w***	6
***b1***	1.5	***l***	6
***b2***	1	***r_x_***	2.5
***c***	0.25	***r_y_***	2.17
***e***	0.5		

**Table 2:** Summary of the effective parameters of the unit cell.

Effective Parameters	Negative Index Frequency Span (GHz)
Permittivity (*ε*)	5–6.21 and 6.75–11.79
Permeability (*µ*)	7.43–15
Refractive Index (*η*)	5.31–5.95 and 7–12.96
DNG	7.43–11.79

**Table 3:** Design parameters of the proposed antenna.

Parameters	Values (mm)	Parameters	Values (mm)
***k***	19	***r_2_***	5.1
***m***	13.6	***s***	2
***n***	23.35	***t***	4
***p***	1.4	***u***	4.61
***q***	3	***v***	9.16
***r_1_***	9	***x***	1

**Table 4:** Comparison of the proposed antenna with previous works.

Ref. No.	Size (mm^2^)	Bandwidth (GHz)	Gain (dB)
[34]	36 × 34	2.68–12.06	−1 to 6.48
[35]	22 × 21	2.9–9.9	−1 to 5
[36]	40 × 40	3.1–10.6	−2 to 6
[37]	21 × 24	3–11.5	3–5.7
[38]	19.36 × 27.72	3.1–15	3.81
Proposed antenna	19 × 23.35	3–20	1.5–3.88 (Realized Gain)
